# Insights into the Molecular Mechanisms and Novel Therapeutic Strategies of Stenosis Fibrosis in Crohn’s Disease

**DOI:** 10.3390/biomedicines13071777

**Published:** 2025-07-21

**Authors:** Yuan Zhou, Huiping Chen, Qinbo Wang, Guozeng Ye, Yingjuan Ou, Lihong Huang, Xia Wu, Jiaxi Fei

**Affiliations:** 1 Department of Gastroenterology, The Sixth Affiliated Hospital, Sun Yat-Sen University, Guangzhou 510655, China; 2 Biomedical Innovation Center, The Sixth Affiliated Hospital, Sun Yat-Sen University, Guangzhou 510655, China; wangqb3@mail.sysu.edu.cn (Q.W.);; 3 Department of Graceland Medical Center, The Sixth Affiliated Hospital, Sun Yat-Sen University, Guangzhou 510655, China; 4 Department of Pharmacy, The Sixth Affiliated Hospital, Sun Yat-Sen University, Guangzhou 510655, China; 5 Department of General Practice, The Sixth Affiliated Hospital, Sun Yat-Sen University, Guangzhou 510655, China

**Keywords:** stenosis, fibrosis, Crohn’s disease, medication, endoscopic, surgery

## Abstract

Crohn’s disease (CD), characterized by chronic gastrointestinal inflammation, is complicated by intestinal stenosis resulting from dysregulated fibrogenesis and is marked by excessive extracellular matrix (ECM) deposition, fibroblast activation, and luminal obstruction. While biologics control inflammation, their failure to halt fibrosis underscores a critical therapeutic void. Emerging evidence highlights the multifactorial nature of stenosis-associated fibrosis, driven by profibrotic mediators and dysregulated crosstalk among immune, epithelial, and mesenchymal cells. Key pathways, including transforming growth factor (TGF-β), drosophila mothers against decapentaplegic protein (Smad) signaling, Wnt/β-catenin activation, epithelial–mesenchymal transition (EMT), and matrix metalloproteinase (MMP) and tissue inhibitors of metalloproteinase (TIMP)-mediated ECM remodeling, orchestrate fibrotic progression. Despite the current pharmacological, endoscopic, and surgical interventions for fibrostenotic CD, their palliative nature and inability to reverse fibrosis highlight an unmet need for disease-modifying therapies. This review synthesizes mechanistic insights, critiques therapeutic limitations with original perspectives, and proposes a translational roadmap prioritizing biomarker-driven stratification, combinatorial biologics, and mechanistically targeted antifibrotics.

## 1. Introduction

Crohn’s disease (CD), a chronic inflammatory bowel disease (IBD) involving transmural discontinuous gastrointestinal inflammation, poses a significant global health challenge. Epidemiological studies have shown the rising incidence of IBD (1.61/100,000 person-years), with CD accounting for 0.34/100,000 cases and demonstrating persistent upward trends worldwide [1]. A hallmark complication of CD is intestinal stricture formation, driven by repetitive cycles of mucosal injury and aberrant tissue repair [2]. Clinically, 20% of patients present with stricturing phenotypes at diagnosis, and > 50% develop symptomatic stenosis during disease progression [3]. Pathophysiologically, stricture development involves dual inflammatory and fibrotic pathways, in which chronic inflammation initiates molecular events culminating in ECM dysregulation. Key mediators (e.g., IL-11, IL-17, IL-34, and TGF-β) activate fibroblast-to-myofibroblast transition, while ROS and PPAR signaling contribute to smooth muscle hyperplasia and intestinal wall distortion [4,5,6].

Despite advances, critical challenges persist in managing fibrostenotic CD: anti-TNF agents and immunomodulators suppress early inflammation-mediated fibrosis [7] but lose efficacy in established fibrotic strictures [8]. Fibrotic progression exhibits substantial heterogeneity, with only a subset of inflammatory lesions evolving into irreversible stenosis, suggesting inflammation-independent regulatory mechanisms (e.g., epithelial–mesenchymal crosstalk, microbiome signals, and mechanotransduction). Current diagnostics lack sensitivity for pre-stenotic changes, and validated biomarkers for progression or therapeutic response are absent.

This study systematically investigates (1) mechanistic reappraisal, which involves synthesizing evidence on mechano-inflammatory pathways, focusing on matrix stiffness modulation of fibroblast activation via integrin signaling and YAP/TAZ translocation; (2) therapeutic critique, critically appraising novel biologics (anti-IL-23p19 and anti-IL-36), small-molecule inhibitors (JAK1/STAT3 and LOXL2), and microbiota-targeted interventions for antifibrotic potential; and (3) a translational framework, proposing a precision medicine framework combining multi-omics, functional imaging, and AI-based modeling to stratify fibrotic risk and guide therapy.

## 2. Histopathological Characteristics of Fibrotic Stenosis in Crohn’s Disease

At diagnosis, 77% of patients with CD present with purely inflammatory pathology, 11% exhibit stricturing behavior, and 16% have fistulizing complications [9]. Longitudinal data show that complication rates rise to 48–52% at 5 years and 69–70% at 10 years post-diagnosis [10]. Intestinal stenosis, which occurs in approximately half of patients with CD, represents one of the most clinically significant complications [11]. With the consistent transmural nature of CD, stenotic lesions involve all intestinal wall layers, featuring marked thickening of the intestinal wall, excessive ECM accumulation, and mesenchymal cell expansion, culminating in a fibrostenotic phenotype underlying clinical obstruction.

## 3. The Mechanism of Intestinal Fibrosis in Crohn’s Stenosis

CD-associated intestinal stenosis involves inflammatory and fibrotic processes, with key features such as extensive collagen, disrupted muscularis mucosae, and fibrotic interstitial thickening [12]. Smooth muscle hyperplasia primarily drives wall thickening, with ileal lesions showing muscular hyperplasia/hypertrophy and colonic lesions exhibiting submucosal/muscularis propria fibrosis.

Creeping fat (hypertrophic mesenteric adipose tissue) promotes progression via subserosal infiltration, muscular layer interaction, and pro-inflammatory mediator secretion [13]. Fibrogenesis involves genetic, environmental, and immune factors through the following pathways: (i) TGF-β-dominated growth factor signaling (IGF, CTGF, and VEGF); (ii) metabolic pathways such as the renin–angiotensin–aldosterone system (RAAS) and mammalian target of rapamycin (mTOR); (iii) immune activation (TLR4 and Th17); (iv) microbial factors (AIEC); (v) fibrinolytic regulation (PAI-1); and (vi) nuclear signaling (PPARγ) [14,15].

**Figure 1:**
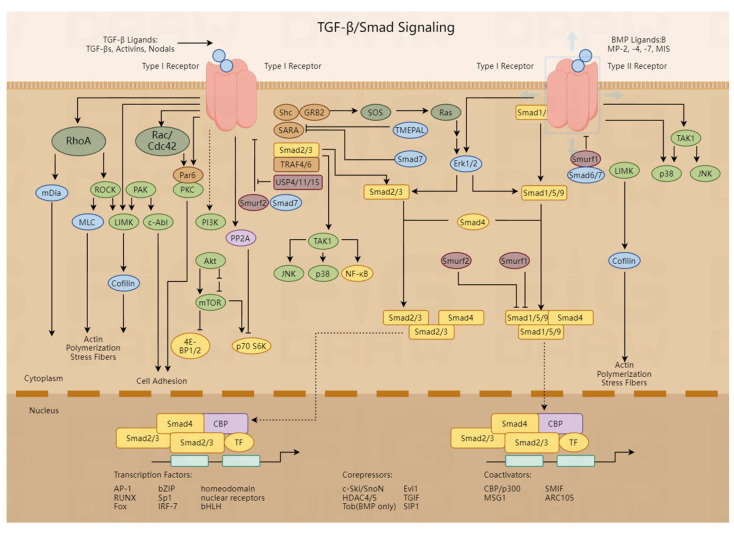
The molecular mechanisms of stenosis fibrosis in Crohn’s disease. The pathogenesis involves the activation of myofibroblasts, dysregulated ECM metabolism, polarization of pro-inflammatory cytokines, and the upregulation of profibrotic mediators such as TGF-β. Additionally, aberrant expression of microRNAs (e.g., miR-29 and miR-200 families) further modulates fibrotic signaling pathways.

These mechanisms synergistically induce myofibroblast activation, creating the diagnostic triad of fibrosis, smooth muscle hypertrophy, and creeping fat expansion (Figure 1, Table 1).

### 3.1. Fibrosis Factors

#### 3.1.1. TGF-β

TGF-β exhibits dual roles. It is anti-inflammatory/fibrinolytic at physiological levels but profibrotic when overexpressed [16]. Among its isoforms, TGF-β1 drives pathological fibrosis; for example, its overexpression in the murine colon induces extensive fibrosis [17]. Sustained TGF-β1 activity exacerbates fibrosis by stimulating ECM secretion, promoting myofibroblast transdifferentiation, disrupting the MMP/TIMP balance to increase net ECM deposition [18], and enhancing profibrotic cytokine secretion. TGF-β1 activates the Smad-dependent, ERK1/2 MAPK, and PKC pathways [19]. MAPK inhibition reduces TGF-β1-induced collagen synthesis in CD patient-derived cells [20], positioning it as a promising antifibrotic target.

#### 3.1.2. IGF

The IGF system contributes to fibrosis via the upregulation of IGF-I, IGFBP-3, and IGFBP-5 in intestinal smooth muscle cells. IGF-I promotes smooth muscle proliferation, suppresses apoptosis, and enhances ECM production. TGF-β mediates intestinal fibrosis partly by elevating IGF-I expression [21]. Cultured smooth muscle cells from CD stenotic segments show IGFBP-3 overexpression, driving COL1A1 upregulation via TGF-βRII/I and Smad2/3 [22].

#### 3.1.3. CTGF

CTGF, induced by cellular mediators in multiple tissues [23], promotes cell proliferation, ECM deposition, and angiogenesis. As a key TGF-β downstream effector, it enhances TGF-β-receptor binding. CTGF is markedly upregulated in CD stenotic tissues (>5-fold vs. normal) [24], exerting effects via the Smad, MAPK, and PKC pathways.

#### 3.1.4. RAAS

RAAS regulates cell growth, ROS production, inflammation, and fibrogenesis [25]. Angiotensin II (Ang II) is elevated in CD, particularly in stricturing subtypes. RAAS inhibition (ACEIs/ARBs) downregulates TGF-β and CTGF [26]. Elevated mucosal lipid peroxides in IBD patients [27] directly implicate oxidative stress in fibrosis pathogenesis.

#### 3.1.5. Other Protein Molecules

In CD stenotic segments, fibroblast-like cells show elevated albumin and N-cadherin expression, facilitating migration. Substance P binding to NK-1R stimulates fibroblast migration via Akt [28]. Hypoxia-induced HIFs enhance integrin-β1 expression, promoting fibroblast contraction and epithelial migration [29]. HSP47 [30], S100A4 [31], and WISP-1 [32] are implicated in fibrosis and EMT.

#### 3.1.6. Upregulation Gene

miR-93-5p shows context-dependent effects; its downregulation in fibrostenotic subserosa suggests a fibroprotective role, mirroring other downregulated fibroprotective miRNAs (miR-133a-3p [34], miR-133b [35], miR-193a-5p [36], miR-335-5p [37], and miR-378a-3p [38]). miR-376c-3p and miR-424-5p are upregulated in fibrostenosis. Genetic loci implicated in CD fibrosis include NOD2/CARD15, DLG5, OCTN transporters, MMP-3 polymorphism, IBD5 variants, ATG16L1, and IL-23R [41]. Mutations in autophagy (ATG16L1 and IRGM) and pathogen recognition genes promote fibrosis via impaired bacterial clearance and sustained cytokine production [42]. While murine TNBS models illuminate TGF-β-driven fibrosis, their translational relevance is constrained by anatomical differences in intestinal wall structure and immune repertoire. Notably, human fibrotic strictures exhibit advanced cross-linked collagen networks rarely replicated in rodents, a key limitation for ECM-targeted therapeutics.

### 3.2. Antifibrotic Factors

#### 3.2.1. mTOR

mTOR regulates cell growth, survival, and protein synthesis. mTOR inhibitors exert antifibrotic effects by reducing fibroblast/myofibroblast populations, suppressing fibrinogen synthesis, and modulating autophagy/angiogenesis [43]. TGF-β/Smad3 directly activates mTOR, enhancing collagen production [44]. Rapamycin/sirolimus blocks TGF-β-induced HIF-1α and fibrosis.

#### 3.2.2. PPAR

Current research has identified several key endogenous antifibrotic factors, including the peroxisome proliferator-activated receptor (PPAR), the Smad7 protein, and adiponectin. Experimental evidence demonstrates that exogenous adiponectin administration markedly attenuates both intestinal inflammation and fibrosis in TNBS-induced murine models, with in vitro studies further revealing its ability to suppress TGF-β1-mediated fibroblast-to-myofibroblast transition, underscoring its therapeutic potential for CD-associated intestinal fibrosis [45]. PPARs, particularly the γ isoform (PPAR-γ), function as nuclear receptors widely expressed in intestinal mucosal cells, including adipocytes, macrophages, and lymphocytes, where they orchestrate diverse biological processes ranging from lipid metabolism and glucose homeostasis to inflammation modulation and fibrogenesis. Mechanistically, PPAR-γ activation exerts potent antifibrotic effects through direct Smad3 inhibition and the subsequent downregulation of CTGF expression within the TGF-β/Smad3 signaling axis [46]. Importantly, therapeutic strategies aimed at enhancing PPAR-γ activity have shown promising results in reducing pathological collagen deposition and ameliorating fibrotic progression, highlighting its central role in intestinal fibrosis regulation.

#### 3.2.3. Adiponectin

Adipocytes in mesenteric adipose tissue function as active endocrine cells, secreting a complex array of both profibrotic and antifibrotic mediators into the circulation, with leptin and adiponectin representing key opposing regulators. Adiponectin inhibits TNF-α signaling, exerting antifibrotic effects [47]. Another adipose-derived factor, C1q/TNF-related protein-3 (CTRP-3), demonstrates significant expression in adipose tissue and is particularly abundant in CD patients. CTRP-3 manifests its antifibrotic activity through a triple mechanism: direct antagonism of TGF-β secretion, downregulation of CTGF expression, and suppression of collagen production [48], positioning it as a promising endogenous modulator of intestinal fibrosis.

#### 3.2.4. Downregulation Gene

MicroRNAs play crucial regulatory roles in fundamental biological processes, including embryonic development, cell differentiation, apoptosis, and proliferation. miR-200b suppresses EMT by downregulating vimentin and upregulating E-cadherin. miR-200b-containing microvesicles attenuate experimental colitis associated intestinal fibrosis by inhibiting epithelial-mesenchymal transition [49]. miR-29b downregulation in fibrostenotic CD targets collagen-producing cells, suppressing collagen I/III synthesis and counteracting TGF-β1-mediated accumulation [50].

### 3.3. Other Factors

#### 3.3.1. Intestinal Microbiota and Intestinal Wall Fibrosis

AIEC invades intestinal epithelium, stimulating pro-inflammatory cytokines (TNF-α, IFN-γ, and IL-17) that drive fibrosis [51]. CD-associated dysbiosis and dysregulated lipid metabolism promote fibrotic markers (α-SMA and vimentin) [52]. Microbiota restoration or TLR/NLR blockade may mitigate fibrosis.

#### 3.3.2. Autophagy

Emerging insights into disease pathogenesis highlight the critical involvement of autophagy-related genes (IRGM, NOD2, and ATG16L1) in CD development, with growing evidence suggesting that autophagy modulation may represent a promising therapeutic strategy for multi-organ fibrosis, including CD-associated intestinal fibrosis [53,54]. Rapamycin attenuates inflammation and reduces fibrosis via autophagy induction [55].

## 4. Therapeutic Strategies for Fibrosis and Crohn’s Disease Stricture

### 4.1. Medications

#### 4.1.1. Conventional Agents

Therapeutic interventions for intestinal fibrosis and stenosis in CD continue to present substantial clinical challenges [56]. Current medical management strategies include 5-aminosalicylic acid (5-ASA) compounds, glucocorticoids, immunosuppressants, and biologic agents, each with distinct limitations in addressing fibrotic complications. While 5-ASA demonstrates minimal efficacy against inflammation-driven fibrosis, corticosteroids can transiently reduce inflammatory edema and obstructive symptoms but are unsuitable for long-term use due to significant adverse effects and an associated increased risk of surgical intervention with prolonged administration.

#### 4.1.2. Biological Agents

Biologics (Table 2) [57,58,59,60,61,62,63,64,65,66,67,68] include anti-TNF-α agents (infliximab, adalimumab, and certolizumab), integrin inhibitors (natalizumab and vedolizumab), and IL-12/23 antagonists (ustekinumab). The STRIDENT trial confirmed anti-TNF efficacy in fibrostenotic CD with optimized dosing [69]. Combination therapy benefits patients with anti-drug antibodies [70]. Real-world data suggest vedolizumab/ustekinumab potential in fibrostenotic CD [71]. Anti-TNF agents reduce hospitalizations [72] and surgical rates (8.4% annual decline alongside 36.2% yearly use increase) [73]. Immunosuppressive/anti-TNF therapy slows progression to stricturing phenotypes [74], stabilizes stenosis-related hospitalizations, and lowers resection rates [75]. Escalation to anti-TNF combinations may delay endoscopic dilation in anastomotic stenosis [76]. Despite reducing the number of surgeries, anti-TNF biologics show minimal impact on established fibrosis due to irreversible ECM remodeling and stromal cell senescence, highlighting a fundamental disconnect between anti-inflammatory and antifibrotic efficacy.

#### 4.1.3. Immunosuppressants

The therapeutic application of immunosuppressants in inflammatory bowel disease traces its origins to 1962 [77], when azathioprine and methotrexate—initially developed for pediatric hematologic disorders by Hitchings and Elion—were first repurposed for IBD management. Some clinical evidence suggests that postoperative azathioprine may modestly delay stenosis recurrence after resection [78]. However, conventional anti-inflammatory therapies minimally impact established fibrosis due to the progressive decoupling of inflammatory and fibrotic processes.

### 4.2. Procedural and Surgical Interventions

Endoscopic balloon dilation (first-line for small bowel), stricturotomy (superior for anastomotic strictures), and stenting (technically successful but high migration risk) offer minimally invasive options for managing Crohn’s disease strictures, though patient selection and technique optimization are crucial (Table 3).

#### 4.2.1. Endoscopic Balloon Dilation (EBD)

Endoscopic balloon dilation (EBD) has emerged as a well-established, minimally invasive alternative to surgical intervention for managing fibrostenotic CD. Supported by extensive observational data, EBD demonstrates both high short-term efficacy and durable long-term outcomes, often serving as a bridge to delay or even avoid the need for surgical resection [79]. In a multicenter study, 69.5% (66/95) of CD patients with small intestinal strictures showed short-term symptomatic improvement post-EBD, with only 6.3% (6/95) requiring surgery [80]. Post-procedural assessment using the Visual Analog Scale (VAS) at four weeks revealed short-term symptomatic improvement in 69.5% of cases (66/95 patients), while only 6.3% (6/95) required subsequent surgical intervention. These findings reinforce that balloon-assisted colonoscopy represents a safe and effective first-line strategy for managing select cases of small bowel strictures in CD, offering significant clinical benefits while minimizing the risks and morbidity associated with repeated surgical procedures.

#### 4.2.2. Stricturotomy

Endoscopic stricturotomy (ES) is effective for CD-related strictures. ES demonstrated superior efficacy vs. EBD for anastomotic strictures, with higher symptomatic (85.7% vs. 64.6%) and endoscopic improvement (90.5% vs. 68.3%) and lower surgical intervention rates (9.5% vs. 33.5%; *p* = 0.03) [82]. These results position ES as a potentially more effective alternative to EBD for managing anastomotic strictures, particularly in cases refractory to conventional dilation techniques, although larger randomized controlled trials are warranted to validate these findings and establish optimal patient selection criteria.

#### 4.2.3. Endoscopic Stent Placement

Endoscopic stenting represents a valuable therapeutic option for managing strictures in CD, with recent systematic reviews showing high technical success rates (93%) but modest clinical success (60.9%) for stenting, with migration rates up to 43.9% [83]. These findings were corroborated by Attar et al. [83] in their evaluation of 46 patients (73.9% with anastomotic strictures), where 93.5% showed initial clinical improvement, though 34.8% (16 patients) required subsequent endoscopic balloon dilation or surgical intervention. While the procedure carries significant risks, including mucosal adhesion (15–20%), perforation (3–5%), and recurrent stent migration (up to 45% in some series), it underscores the need for further research to optimize patient selection, stent design, and procedural techniques to enhance the risk–benefit profile of endoscopic stenting in CD stricture management.

## 5. Future Perspectives and Treatment of Stenosis Fibrosis in Crohn’s Disease

Despite anti-inflammatory advances, intestinal fibrosis management remains challenging (Table 4). Persistent stenosis/surgery rates despite biologics highlight this gap [87,88,89]. Promising therapeutic targets include: (1) The WNT/β-catenin pathway, where the inhibitor ICG-001 attenuates TGF-β-mediated fibrogenesis in preclinical models [90]; (2) Rho kinase (ROCK) signaling, with localized ROCK inhibitors (e.g., AMA0825) suppressing myofibroblast activation and ECM deposition via non-canonical TGF-β pathways [91]; (3) PPAR-γ activation, which dually inhibits both WNT/β-catenin and TGF-β signaling [92]; and (4) Amniotic epithelial cells (AECs), exhibiting multimodal antifibrotic, anti-inflammatory, and regenerative effects in IBD models through cytokine modulation [93]. While these strategies demonstrate compelling preclinical potential, rigorous clinical validation is imperative to translate findings into practice, emphasizing the need for well-designed trials targeting fibrosis-specific pathways in CD.

### 5.1. ECM Dysregulation

MMP/TIMP imbalance and excessive mesenchymal proliferation drive ECM accumulation. Elevated TIMP1 and LOX enhance ECM stiffness; LOX inhibition may attenuate fibrosis. FAP suppression reduces collagen I/TIMP1 [99]. Pirfenidone inhibits α-SMA, MMP3, and collagen I in CD myofibroblasts [100].

### 5.2. TGF-β/Smad and Antioxidant Pathways

TGF-β1 drives ECM production; Smad2/3 deficiency mitigates fibrosis. TGF-β/Smad signaling suppresses Nrf2, elevating ROS and amplifying fibrosis [101]. The Nrf2 activator tBHQ downregulates TGF-β1/Smad2/3/P-Smad2/3 [102]. PPAR-γ agonists inhibit Smad2 and reduce TGF-β1-induced α-SMA [103]. Smad7 restoration reduces TGF-β1, P-Smad3, and collagen deposition [104].

### 5.3. Targeting EMT

EMT contributes to fibrosis via the epithelial-to-mesenchymal transition. Curcumin upregulates PPAR-γ/E-cadherin and suppresses α-SMA. Wumei pill components (citric acid, coptisin, and ginsenoside Rb1) inhibit fibroblast proliferation and EMT [105].

## 6. Conclusions

Stenosis fibrosis in CD arises from intricate interactions among immune cells, fibroblasts, and epithelial cells. Key pathways, including TGF-β, Wnt/β-catenin, IL-13, IL-17, and Hedgehog signaling, drive fibrogenesis. To address this unmet clinical need, future efforts should prioritize biomarker discovery for early fibrosis detection, clinical evaluation of novel antifibrotic agents (e.g., pirfenidone and ROCK/LOX inhibitors), and mechanistic studies to refine pathway-specific interventions.

## Figures and Tables

**Table 1:** Main molecular pathways and key factors involved in the fibrosis of Crohn’s disease.

	Molecular Pathway	Main Factors	Mechanism	Therapeutic Targeting and Potential
Fibrosis factors [16,17,18,19,20,21,22,23,24,25,26,27,28,29,30,31,32,33,34,35,36,37,38,39,40,41,42]	TGF-β, Smad	TGF-β1, TGF-β2, TGF-β3, Smad2, and Smad3	Activates myofibroblasts and promotes ECM synthesis.	High (targeting TGF-β or Smad3)
	IGF-I, Smad2/3, Smad, MAPK, PKC	IGFBP-3,CTGF, IGFBP-5, TGF-βRII/I, Smad2/3, and COL1A1	The IGF system contributes significantly to fibrosis development in CD patients, primarily through the upregulation of IGF-I, IGFBP-3, and IGFBP-5 in intestinal smooth muscle cells.	High (targeting TGF-β or Smad3)
	ROS, ECM	ECM, Angiotensin II, ARBs, and ACEIs	Regulating ganglionic cell growth, differentiation, proliferation, and apoptosis, while also influencing ROS production, cytokine expression, endothelial cell activation, inflammatory responses, ECM synthesis, and fibrogenesis.	High (targeting ROS and ECM)
	NK-1R	HSP47, NK-1R, and WISP-1	Inflammatory-induced tissue hypoxia upregulates HIFs, which selectively enhance integrin-β1 expression in fibroblasts, thereby promoting fibroblast contraction and epithelial cell migration, a potential mechanism underlying intestinal fibrosis development in CD; heat shock protein 47 (HSP47), implicated in inflammatory bowel disease-associated fibrosis, has emerged as a promising therapeutic target.	New (targeting HSP47, NK-1R, and WISP-1)
	Upregulation gene	MiR-93-5p, NOD2/CARD15, DLG5, OCTN-1/2, MMP-3, ATG16L1, and IL-23R	The deposition of ECM increases while the degradation decreases, gene expression reprogramming, and profibrotic phenotype.	High (targeting specific collagens or TIMPs)
Antifibrotic [43,44,45,46,47,48,49,50]	PI3K/Akt	PIKK family, mTOR, and TGF-β/Smad3	The TGF-β/Smad3 pathway can directly activate mTOR signaling, creating a positive feedback loop that enhances collagen production and fibrotic progression.	High (targeting TGF-β or Smad3)
	TGF-β, Smad3	PPAR, Smad7 protein, and adiponectin	Through lipid metabolism and glucose homeostasis to adjust inflammation modulation and fibrogenesis.	High (targeting TGF-β or Smad3)
	Downregulation gene	miR-200b, vimentin, and miR-29	MiR-29b can counteract TGF-β1-mediated collagen accumulation, highlighting its potential as a therapeutic target for intestinal fibrosis.	New (targeting TGF-β or Smad3)
Other factors [50,51,52,53,54,55]	Oxygen radicals, lipid peroxides	ROS and ECM	Contributes to ECM deposition, fibroblast activation, and sustained inflammatory responses that drive fibrotic remodeling.	New (targeting ROS and ECM)
	Intestinal microenvironment harbors	TNF-α, IFN-γ, PRRs, NLRs, TLRs, AIE, IL-6, IL-23, IL-17, and IL-10	Immune dysregulation leads to the formation of a pro-inflammatory microenvironment.	Medium (targeting IL-6/23)
	Autophagy	IRGM, NOD2, and ATG16L1	Attenuates intestinal inflammation through immunosuppression and reduces fibrotic progression via autophagy induction.	New (targeting IRGM, NOD2, and ATG16L1)

PIKK: phosphoinositide 3-kinase-related kinase; PPAR: peroxisome proliferator-activated receptor; EMT: epithelial–mesenchymal transition; TNF-α: tumor necrosis factor-α; Th1: type 1 T helper cell; CTRP-3: C1q/TNF-related protein-3; PRRs: pattern recognition receptors, TLRs: toll-like receptors; NLRs: NOD-like receptors; AIEC: adherent-invasive Escherichia coli; ROS: reactive oxygen species; ACEIs: angiotensin-converting enzyme inhibitors; ARBs: angiotensin II receptor blockers; NK-1R: neurokinin-1 receptor; HSP47: heat shock protein 47; WISP-1: WNT1-inducible signaling pathway protein-1; OCTN-1/2: OCTN transporters.

**Table 2:** Biological agents for Crohn’s treatment.

Drug Name	Key Clinical Findings in Fibrostenotic CD	Category	Approved	Usage	References
Infliximab	56% reduction in surgery risk vs. placebo (HR 0.44; *p* = 0.02), 32% stricture improvement on MRI at 1 year	Anti-TNF-ɑ	Remicade CD (1998, FDA; 1999, EMA)	IV/SC (the initial dose is 5 mg/kg, given once in weeks 2 and 6 and then once every 8 weeks).	[57,58,59]
Adalimumab	41% decreased hospitalization for obstruction, median time to surgery of 2.8 years vs. 0.9 years (untreated)	Anti-TNF-ɑ	Humira CD (2007, FDA; 2006, EMA)	SC (the initial dose is 80 mg each time, followed by an injection of 40 mg in the second week. Then, 40 mg injections are given every one or two weeks to maintain the therapeutic effect).	[60,61,62]
Vedolizumab	68% clinical response in anti-TNF refractory strictures, 48% reduced endoscopic progression at 52 weeks	Integrin inhibitor	Entyvio CD (2014, FDA; 2014, EMA)	IV/SC (an intravenous infusion of 300 mg was administered every 30 min during weeks 0, 2, and 6, and then every 8 weeks).	[63,64,65,66]
Ustekinumab	63% symptomatic improvement in complex strictures, 3.1-fold lower resection rate vs. conventional therapy	Anti-il-12/23 monoclonal antibody	Stelara CD (2016, FDA; 2018, EMA)	Body weight less than or equal to 55 kg for the first dose of 260 mg; body weight greater than 55 kg and less than or equal to 85 kg for the first dose of 390 mg; body weight less than or equal to 55 kg for the first dose of 260 mg; weights greater than 85 kg were given 520 mg for the first dose, 90 mg after 8 weeks, and 90 mg after 12 weeks	[67,68]

CD: Crohn’s disease; UC: ulcerative colitis; IV: intravenous injection; SC: subcutaneous injection.

**Table 3:** Surgery strategy for fibrosis and stenosis in Crohn’s disease.

Surgical Intervention	Author, Year	Study Type	Total Number (185)	Control Group (*n*)	Observation Group (*n*)	*p*-Value	Median Year
Endoscopic Balloon Dilation (EBD)	Bettenworth D, 2017 [78]	Retrospective study	1463	N/A	N/A	0.008	N/A
	Hirai F, 2018 [79]	Randomized, controlled, open-label, multicenter trial	112	11	95	<0.001	Short-term outcomes (4 weeks); long-term outcomes (2 years)
Stricturotomy	Mohy-Ud-Din N, 2020 [80]	Review	12	11	1	N/A	N/A
	Lan N, 2018 [81]	Observation study	185	21	61	0.03	0.8 (IQR: 0.1–1.6) year and 4.0 (IQR: 0.8–6.9)
Endoscopic Stent Placement	Chandan S, 2023 [82]	Review	163	N/A	N/A	N/A	Pooled rate of clinical success: 60.9% (95% confidence interval [CI], 51.6–69.5); I2 = 13%); technical success: 93% (95% CI, 87.3–96.3; I2 = 0%).
	Attar A, 2021 [83]	Pembrolizumab	46	27	19	N/A	The overall success rate: 58.7% [n = 27]; median follow-up of 26 months [8–41 months]
Operation treatment	Ponsioen C, 2017 [84]	Randomized, controlled, open-label, multicenter trial	143	73	70	0.25	12 months
	Bemelman WA, 2018 [85]	Consensus	N/A	N/A	N/A	N/A	N/A
	Bislenghi G, 2022 [86]	Review	1839	N/A	N/A	N/A	Postoperative complication rates: 15.5% [95% CI 11.2–20.3%]; 7.4% [95% CI 0.2–22.9%]; and 19.2% [95% CI 5–39.6%]

**Table 4:** Therapeutic approaches for fibrostenotic CD: efficacy, limitations, and future directions.

Agent/Approach	Mechanism	Current Efficacy Evidence	Key Limitations	Future Optimization
Anti-TNF biologics	TNF-α neutralization	Surgery rates (36.2% annual use) [94]; STRIDENT trial: symptom relief	No fibrosis reversal; ECM irreversibility	Combinatorial PPAR-γ agonists
Vedolizumab	α4β7 integrin blockade	Real-world: fistula closure (34%); stricture data pending [95]	Limited stricture-specific trials	Phase III trials for fibrotic endpoints
PPAR-γ agonists	Dual TGF-β/Wnt inhibition	Preclinical: collagen I, α-SMA [96,97]	Systemic toxicity (rosiglitazone)	Gut-targeted delivery systems
Pirfenidone	TGF-β, collagen synthesis	CD myofibroblasts: α-SMA, collagen I (0.5–2 mg/mL) [98]	Limited human data; dosing uncertainty	Localized colonic formulations
Endoscopic stricturotomy	Mechanical stricture release	90.5% technical success; surgery vs. EBD (9.5% vs. 33.5%) [84]	Expertise-dependent; long-term durability	Standardized training protocols

## Data Availability

Not applicable.

## Abbreviations

IBD: inflammatory bowel disease; CD: Crohn’s disease; UC: ulcerative colitis; TNF-α: anti-tumor necrosis factor-α; TGF: transforming growth factor; Smad: drosophila mothers against decapentaplegic protein; ECM: extracellular matrix; ROS: reactive oxygen species; PPARs: peroxisome proliferator-activated receptors; IGF: insulin-like growth factor; CTGF: connective tissue growth factor; RAAS: renin–angiotensin–aldosterone system; mTOR: mammalian target of rapamycin; VEGF: vascular endothelial growth factor; 5-ASA: 5-aminosalicylic acid.
